# SARS-CoV-2 vaccines induce a diverse spike-specific CD4+ T cell receptor repertoire in people living with HIV with low CD4 nadirs

**DOI:** 10.3389/fimmu.2025.1663819

**Published:** 2025-10-13

**Authors:** Alicia Mercado, Joel Sop, Steven Amanat, Li Zhang, Natasha M. Chida, Christie R. Basseth, Kelly A. Gebo, Annukka A. R. Antar, Kellie N. Smith, Zhen Zeng, Joel N. Blankson

**Affiliations:** ^1^ Department of Medicine, Johns Hopkins Medicine, Baltimore, MD, United States; ^2^ Bloomberg∼Kimmel Institute for Cancer Immunotherapy, Johns Hopkins Medicine, Baltimore, MD, United States; ^3^ Sidney Kimmel Comprehensive Cancer Center, Johns Hopkins Medicine, Baltimore, MD, United States; ^4^ Department of Molecular and Comparative Pathobiology, Johns Hopkins Medicine, Baltimore, MD, United States

**Keywords:** CD4 T cell, SARS-CoV-2, HIV, T cell receptor (TCR), common cold coronaviruses

## Abstract

People living with HIV with low CD4 T cell nadirs on antiretroviral therapy have suboptimal responses to immunization. We analyzed the SARS-CoV-2 spike-specific CD4+ T cell repertoire in individuals with CD4 nadirs of less than 100 cells/ul who received a primary SARS-CoV-2 mRNA vaccine series as well as the bivalent ancestral/BA.5 spike mRNA vaccine. We tested the hypothesis that antigenic imprinting would result in the preferential expansion of pre-existing cross-reactive T cells that were primed against the 4 common cold coronaviruses. We found that these individuals made robust effector and memory T cell responses to the SARS-CoV-2 spike protein that exceeded the responses to spike proteins from the common cold coronaviruses. Furthermore, in 4 individuals, the number of SARS-CoV-2 specific TCRs far exceeded the number of common cold coronavirus-specific T cell receptors. TCRs that were cross-reactive for common cold coronaviruses and SARS-CoV-2 comprised less than 10% of the total detected SARS-CoV-2 specific T cells. The diversity of the SARS-CoV-2 spike-specific repertoire in 6 study participants was comparable to that of the repertoire in vaccinated HIV healthy donors. Our data suggests people living with HIV with low CD4 nadirs can have significant functional immune reconstitution with little evidence of antigenic imprinting due to pre-existing T cell responses to common cold coronaviruses.

## Introduction

People living with HIV (PLWH) with low CD4 nadirs have limited responses to immunization with antigens and some vaccines (1, 2). Lange et al. found that the CD4 nadir predicted T cell responses to immunization with tetanus toxoid, diphtheria-toxoid, and keyhole limpet hemocyanin in PLWH on antiretroviral therapy (1). Similarly, Tebas et al. found that PLWH with low CD4 nadirs on antiretroviral therapy were less likely to respond to an H1N1 vaccine (2). The mechanisms responsible for this are unknown but disruptions in the T cell receptor (TCR) repertoire that are not fully restored with antiretroviral therapy have been reported in these individuals (3). The presence of a restricted naïve TCR repertoire could potentially lead to the phenomenon of antigenic imprinting which is also called the original antigenic sin. The latter term was coined by Thomas Francis in 1960 to describe the observation that infection with a new strain of Influenza boosted the antibody responses against strains of the virus that an individual had been previously exposed to (4). A similar phenomenon has been observed with T cell responses (5), and recent observations suggest that in some cases, the expansion of pre-existing cross-reactive responses can come at the expense of the development of mono-reactive responses. This is important as mono-reactive T cells may have higher affinity for the novel antigen they are primed against (6, 7). Many studies have shown some degree of pre-existing T cell immunity to SARS-CoV-2 due to cross recognition of SARS-CoV-2 by T cells that were primed against the 4 common-cold coronaviruses (8). In this study we tested the hypothesis that patients with low CD4 nadirs with variable degrees of immune reconstitution on antiretroviral therapy would show evidence of antigenic imprinting and thus would not have a robust mono-reactive T cell response to immunization with SARS-CoV-2 mRNA vaccines. We achieved this by using the functional expansion of specific T cells (FEST) assay to compare the T cell receptor repertoire after stimulating peripheral blood mononuclear cells (PBMCs) with spike protein from SARS-CoV-2 or the 4 common cold coronaviruses (7–12). This assay sequences the CDR3 region of the beta chain of the T cell receptor (TCR) of cells that have been cultured with antigens and therefore can identify expanded antigen-specific clones (13, 14). It can also distinguish between TCRs that cross-recognize SARS-CoV-2 and common cold coronavirus spike proteins versus those that are mono-reactive for a specific spike protein (7). Our data suggest that the SARS-CoV-2 specific T cell response in PLWH with low CD4 nadirs was mostly mono-reactive in nature. Thus, antigenic imprinting does not appear to play a major role in the T cell responses to SARS-CoV-2 in these patients.

## Methods

### Study participants

The study was approved by the Johns Hopkins University Institutional Review Board. Written informed consent was obtained from all participants prior to their inclusion in the study. The clinical characteristics of the study participants studied are summarized in **Supplementary Table 1**. The healthy donors were described in a prior study (11). Blood for the initial SARS-CoV-2 ELISpot and FEST assays was drawn a median of 189 days after receipt of the bivalent ancestral spike/BA.5 spike mRNA vaccine (range 127–278 days) and the participants had a median age of 45 years (range 29–57 years). The PLWH had blood drawn a median of 174 days post vaccination (range 54 to 307 days) and had a median age of 55 years (range 37 to 63 years). The median CD4 nadir was 36 cells/ul (range 1-90). One study participant (CP100) had a CD4 nadir of 2 cells/ul and prolonged SARS CoV-2 shedding prior to initiating ART (15). For the SARS-CoV2 and common cold coronavirus FEST assays, blood was obtained a median of 436 days after the bivalent ancestral spike/BA.5 spike mRNA vaccine was given (range 426–487 days, **Supplementary Table 2**). CP100, had also received the monovalent XBB1.5 vaccine 106 days prior to the blood draw.

### Serology

Multi-array electrochemiluminescence detection technology from MesoScale Diagnostics V-Plex SARS-CoV-2 Panel 31 were used to evaluate IgG binding antibodies to SARS-CoV-2 spike protein in a prior study (11). PLWH with low CD4 nadirs were also tested for antibodies against HCoV-NL63, HCoV-OC43, HCoV-229E, and HCoV-HKU1. Antibody responses were evaluated using ELISA kits purchased from Alpha Diagnostics International following the manufacturer’s instructions as previously described (9).

### Peptides and ELISpot assays

**Figure 1:**
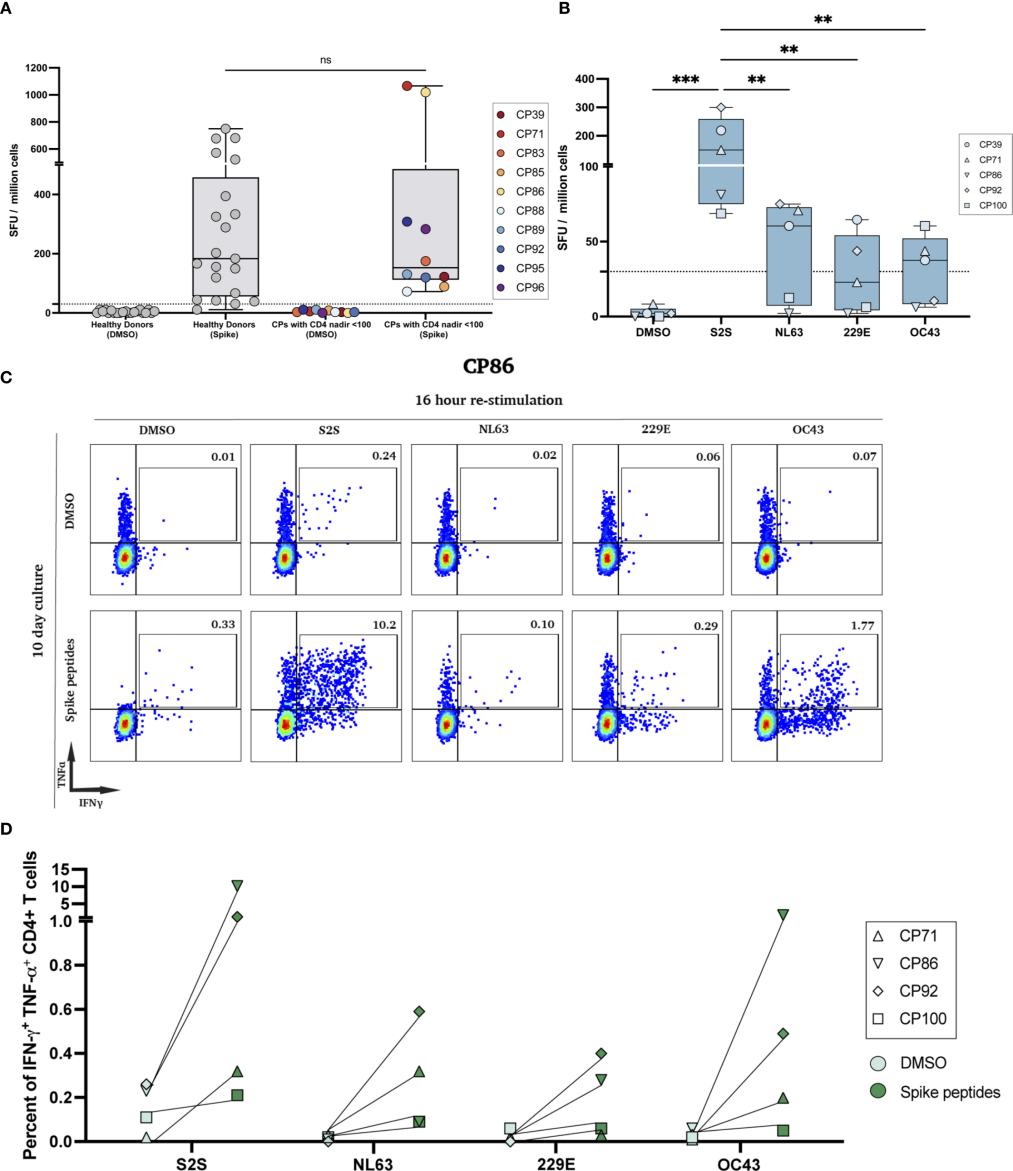
SARS-CoV-2 ELISpot responses and polyfunctional responses after peptide expansion. **(A)** IFN-γ ELISpot with the SARS-CoV-2 spike peptide pool (S2S) on PBMCs from HIV-seronegative donors and PLWH with historical CD4 nadir <100; DMSO controls shown for each cohort (ns, not significant). **(B)** Low-nadir CP IFN-γ ELISpot responses to DMSO, S2S, and common-cold coronavirus pools (HCoV-NL63, -229E, -OC43). (**p<0.01, ***p<0.001). **(C)** Representative flow cytometry plots for CP86. Day-10 spike-expanded CD4^+^ T-cells were re-stimulated for 16 hours with DMSO (top row) or the indicated peptide pools (bottom row); numbers indicate % IFN-γ^+^TNF-α^+^ of CD4^+^ T cells. **(D)** Quantification of IFN-γ^+^TNF-α^+^ CD4^+^ T cells across low-nadir CPs after 7-day (CP71, CP100) or 10-day (CP86, CP92) culture with each peptide pool vs DMSO. Each pair represents the same participant; lines connect paired conditions.

The ELISpot data in **Figure 1A** were previously obtained (11). The SARS-CoV2 ancestral spike peptide pool consisted of a pool of 315 peptides derived from 15 mers with 11 amino acid overlaps obtained from JPT Peptide Technologies. Peptide pools for the spike proteins of HCoV-NL63, HCoV-229E, HCoV-OC43, and SARS-Cov-2, shown in **Figure 1B** were obtained from BEI Resources and were reconstituted with DMSO at a concentration of 10 mg/mL. The HCoV-229E S protein peptide pool has 195 peptides consisting of 17 mer with 11 amino acid overlaps. The HCoV-NL63 S protein peptide pool has 226 peptides made up of 14–17 mer with 11–13 amino acid overlaps. The HCoV-OC43 S protein peptide pool has 226 peptides made up of 17 or 18 mer with 11 amino acid overlaps. The SARS-CoV-2 peptides are 12 mer, 13 mer, or 17 mer, with 10 amino acid overlaps. IFN-γ ELISpot assays were performed as previously described (11). Briefly ELISpot Pro and ELISpot Plus kits with precoated plates were purchased from Mabtech. The wells were plated with unfractionated PBMCs or CD8+ T cell–depleted PBMCs at 130,000-250,000 cells/well, and the cells were cultured for 20 hours with HCoV peptides at a concentration of 1 μg/mL. The plates were then processed according to the manufacturer’s protocol and read by a blinded independent investigator using an automated reading system.

### Expansion culture assay

PBMCs were cultured in RPMI with 10% fetal calf serum with 10 U/mL IL-2 and raltegravir (4uM) and 1 μg/mL of peptide pools or DMSO for either 7 (CP71, CP100) or 10 days (CP86, CP92) as previously described (9). Half of the media was removed and replaced with fresh media with 10 U/ml IL2 on day 3 and day 7. The cells were then washed and replated in fresh media and rested for 6 hours before they were stimulated again with 5 μg/mL of either the same peptide pool or DMSO with protein transport inhibitors (GolgiPlug, 1 μg/mL; GolgiStop, 0.7 μg/mL) and 1ug/ml of antibodies against CD28 and CD49d (all from BD Biosciences). After a 16-hour incubation, the cells were washed and stained with antibodies against CD3 (APC-Cy-7, BioLegend), and CD4 (PerCP-CY-5.5, BioLegend). The cells were then fixed, permeabilized, and stained intracellularly for TNF-α (PE-Cy-7, BD Biosciences, 557647) and IFN-γ (APC, BD Biosciences).

### FEST assay

The FEST assay utilized ancestral SARS-CoV-2, HCoV-NL63, HCoV-229E and HCoV-OC43 spike peptide pools from BEI resources (NIAID, NIH) as well as HCoV-HKU1 spike peptides from JPT Peptide Technologies (Berlin, Germany), to activate CD8+ T cell-depleted PBMCs from the 4 participants as previously described. One participant, CP88, had 2 assays performed. One with SARS-CoV-2, HCoV-NL63, HCoV-229E and HCoV-OC43 spike peptides, and another with SARS-CoV-2 and HCoV-HKU1 spike peptides alone. All peptide pools were used at a concentration of 1 ug/ml. On day 10, cells were harvested, and DNA was extracted using the QIAmp micro-DNA kit (QIAGEN). TCR-Seq was conducted at the Johns Hopkins FEST and TCR Immunogenomics Core Facility (FTIC) using the Ampliseq TCR Beta Short-Read Assay, sequenced on the Illumina sequencer platform (iSeq100, MiSeq and NextSeq1000) with unique dual indexes as previously described (7). Data was uploaded to the MANAFEST analysis tool (http://www.stat-apps.onc.jhmi.edu/FEST/) to identify antigen-specific T cell clonotypes. Positive responses were required to have a mean frequency of greater than 0.1% in at least two replicates, with at least a 5-fold increase over the DMSO controls. Mono-reactive responses were identified if these criteria were met and the mean frequency was 5-fold higher than responses to other spike proteins. Individual receptors analyzed are detailed in **Supplementary Table 3**.

### Spike-specific repertoire diversity

From the three replicate experiments performed for each patient in the FEST assay, frequencies of spike-specific clonotypes were normalized to the spike-specific subset. Shannon’s diversity index (log2 base) was calculated for each individual replicate of each patient. Then, the indices for each patient were averaged and the patient groups were compared using the Mann-Whitney U test. P < 0.05 was considered statistically significant. It is noted that Shannon’s diversity index is a metric conventionally used for entire TCR repertoires, while it is used here to compare antigen-specific subsets.

### Levenshtein distances for sequence homology

The spike-specific TCR sequences for the two patient groups were pooled together to assess homology across groups. To obtain the non-redundant region of the TCR sequences, the first three and last three amino acids were removed from the TCR Vβ CDR3 sequence. Then, the Levenshtein distances were computed for every pair of TCR sequences across the pooled dataset using the stringdist R package (16). An unrooted phylogenetic tree was generated to visualize sequence homology using the ape R package, with each leaf representing a TCR Vβ CDR3 sequence (17). From the overall tree, branches were manually selected by node number and visualized as heatmaps using the pheatmap R package (18). In the heatmaps, each row represented a TCR Vβ CDR3 sequence and the color scale was fixed across maps. All analyses were performed using R version 4.4.2.

## Results

### PLWH with low CD4 nadirs have robust SARS-CoV-2-specific effector and memory T cell responses

In a prior study, we used the IFN-γ ELISpot assay to measure the frequency of ancestral SARS-CoV-2 spike-specific effector T cell responses in PLWH and healthy donors after they received the bivalent ancestral/BA.5 spike mRNA vaccine (11). As shown in **Figure 1A**, there was no significant difference in the frequency of effector T cells in the 2 groups of study participants. In order to compare the frequency of effector T cells that recognized spike peptides from SARS-CoV-2 versus 3 of the 4 common cold coronaviruses, we again performed an ELISpot assay. As shown in **Figure 1B**, the frequency of ancestral SARS-CoV-2 spike peptide-specific T cells was significantly higher than the frequency of T cells specific for spike peptides from HCoV-OC43, HCoV-NL63, and HCoV-229E.

To determine the frequency of memory T cells that recognized the spike peptides from each virus, we performed an expansion assay where PBMCs were cultured with either DMSO or spike peptide pools from each virus for 7 to 10 days and then restimulated the cells for 16 hours with the same peptide pool. As shown for CP86 in **Figure 1C**, there was an expansion of SARS-CoV-2 and HCoV-OC43 spike-specific memory CD4+ T cells that co-expressed IFN-γ and TNF-α. However, the frequency of the SARS-CoV-2 specific memory cells was 5-fold greater. In all 4 participants tested, the frequency of SARS-CoV-2 spike-specific memory CD4+ T cells was higher than the frequency of memory CD4+ T cells specific for the common cold coronavirus spike peptides (**Figure 1D**).

### The number of detected TCRs specific for SARS-CoV-2 greatly exceeds the number of TCRs specific for the common cold coronavirus in PLWH with low CD4 nadirs

**Figure 2:**
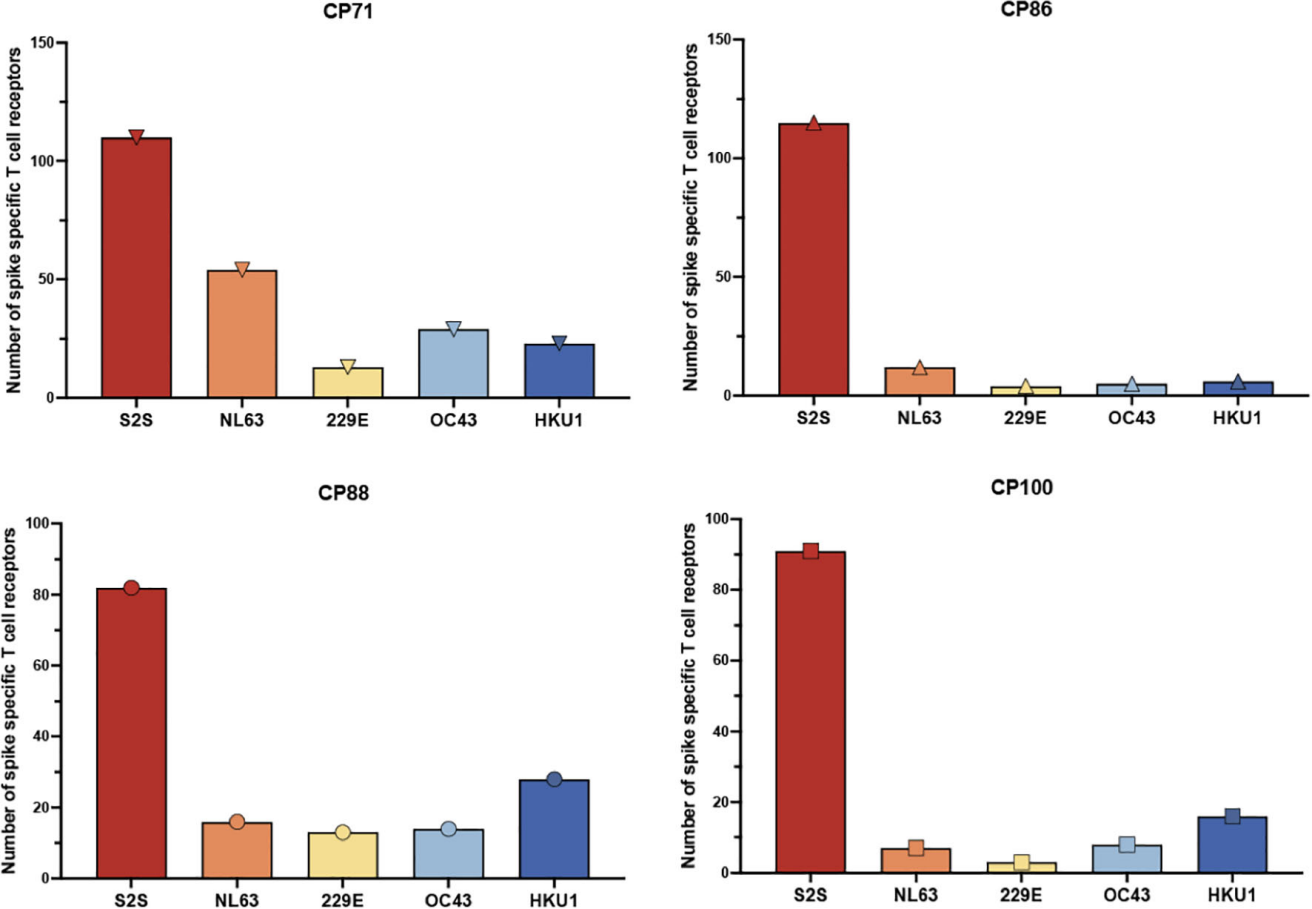
Frequency of spike-specific CD4^+^ T cell receptors recognizing SARS-CoV-2 and common cold coronavirus peptides. The total number of spike-specific CD4^+^ TCR clonotypes identified by the FEST assay is shown for four CPs with low CD4 nadirs. TCRs were classified based on their expansion following stimulation with spike peptide pools derived from SARS-CoV-2 (S2S) or common cold human coronaviruses HCoV-NL63, HCoV-229E, HCoV-OC43, and HCoV-HKU1. Each bar represents the total number of unique spike-specific TCRs detected per condition, with error bars indicating standard deviation across three technical replicates.

The ELISpot and expansion assays measure the frequency of the antigen-specific T cells but not the breadth of the response. In order to measure this parameter, we performed the FEST assay to determine the breadth of TCRs that were specific for spike proteins from SARS-CoV-2 versus the common cold coronaviruses. As seen in **Figure 2**, the number of total SARS-CoV-2 specific TCRs detected ranged from 82 to 115 TCRs with a median of 100.5 TCRs. In contrast, the numbers of TCRs specific for each of the common cold coronaviruses detected ranged from 7 to 54 TCRs with a median of 14 TCRs for HCoV-NL63, 3 to 13 TCRs with a median of 8.5 TCRs for HCoV-229E, 5 to 29 TCRs with a median of 11 TCRs for HCoV-OC43, and 6 to 28 TCRs with a median of 19.5 TCRs for HCoV-HKU1.

### The percentage of SARS-CoV-2 spike mono-reactive TCRs greatly exceeds those of SARS-CoV-2 and common cold coronavirus cross-reactive TCRs

**Figure 3:**
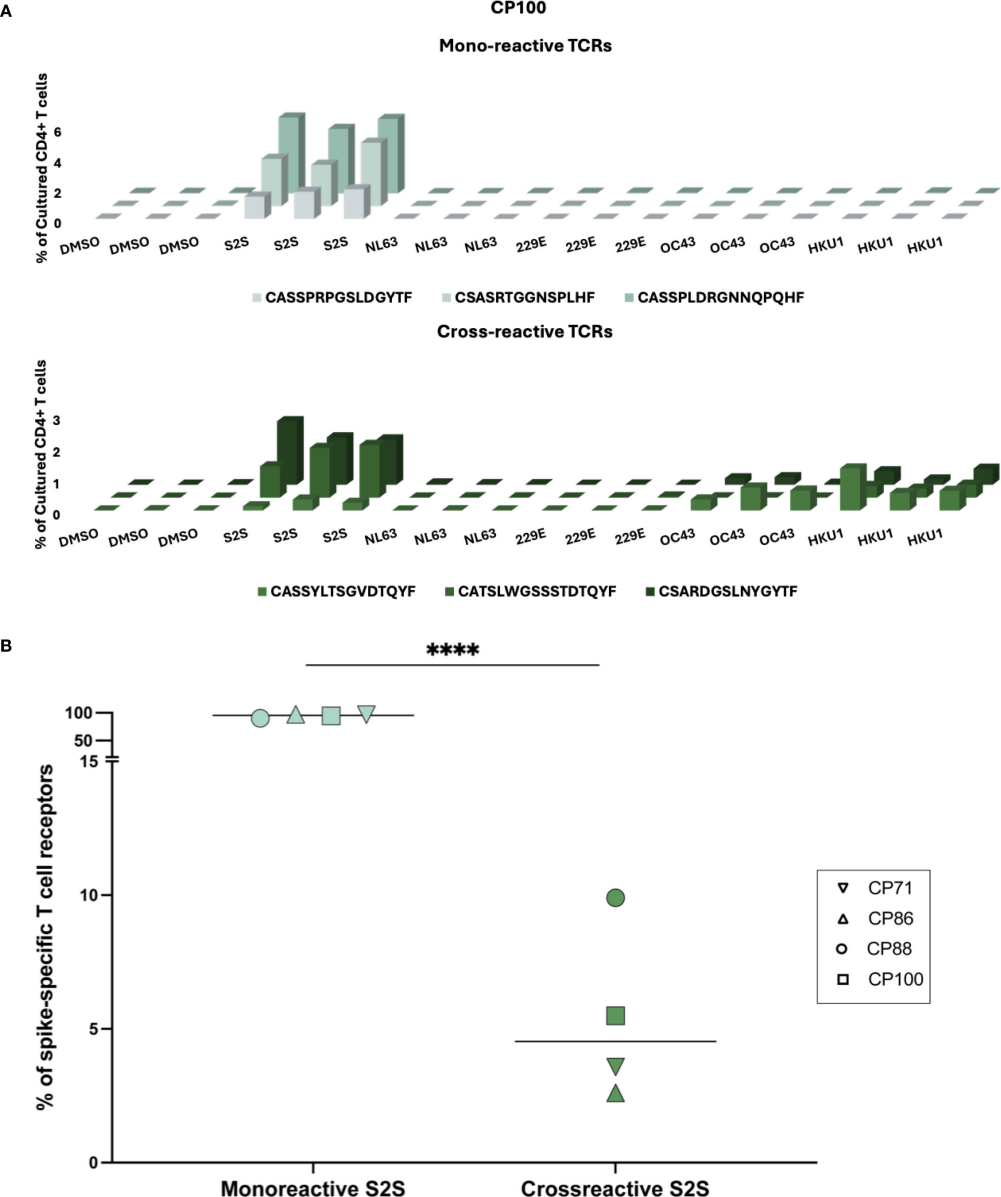
Cross-reactivity of spike-specific CD4^+^ TCRs following in vitro peptide stimulation. **(A)** Representative expansion profiles of three mono-reactive (CASSPRPGSLDGYTF, CSASRTGGNSPLHF, CASSPLDRGNNQPQHF) and three cross-reactive (CASSYLTSGVDTQYF, CATSLWGSSSTDTQYF, CSARDGSLNYGYTF) CD4^+^ TCR clonotypes from participant CP100. TCRs were identified using the FEST assay. The frequencies (% of cultured CD4^+^ T cells, y-axis) of distinct TCR clonotypes (z-axis) across peptide conditions (x-axis), including SARS-CoV-2 spike (S2S) and common cold coronavirus (HCoV-NL63, HCoV- 229E, HCoV-OC43, HCoV-HKU1) peptide pools. Three technical replicates were performed for each condition. **(B)** Quantification of the proportion of mono-reactive versus cross-reactive S2S-specific CD4^+^ TCR clonotypes for four CPs. Horizontal lines indicate group medians. Mono-reactive TCRs were defined as those expanding only in response to S2S peptides, while cross-reactive TCRs expanded to both S2S and at least one common cold coronavirus peptide pool. ****p < 0.0001.

Functional assays cannot distinguish between individual T cells with receptors that cross-recognize different antigens versus separate populations of T cells that recognize each antigen. Thus, we used the FEST assay to determine the frequency of TCRs that cross-recognized spike peptides from SARS-CoV-2 and the common cold coronaviruses. We identified TCRs that recognized SARS-CoV-2 spike alone and others that cross-recognized SARS-CoV-2 and common cold coronavirus spike peptides in each participant. Three representative mono-reactive and cross-reactive TCRs are each shown for CP100 in **Figure 3A**. Across all 4 participants, the percentage of mono-reactive TCRs (median of 96.4%, range from 90.1% to 97.4%) greatly exceeded that of cross-reactive TCRs (median of 4.5% range from 2.6% to 9.9%) (**Figure 3B**). Of the cross-reactive TCRs specific for SARS-CoV-2 and at least 1 common cold coronavirus, the majority cross-recognized SARS-CoV-2 and HKU1 spike peptides (**Supplementary Figure 2**). There were a few TCRs that cross-recognized spike peptides from SARS-CoV-2 and 2 or more common cold coronaviruses (**Supplementary Figure 2**).

### Diversity of the SARS-CoV-2 spike-specific TCRs is similar to that seen in vaccinated healthy donors

**Figure 4:**
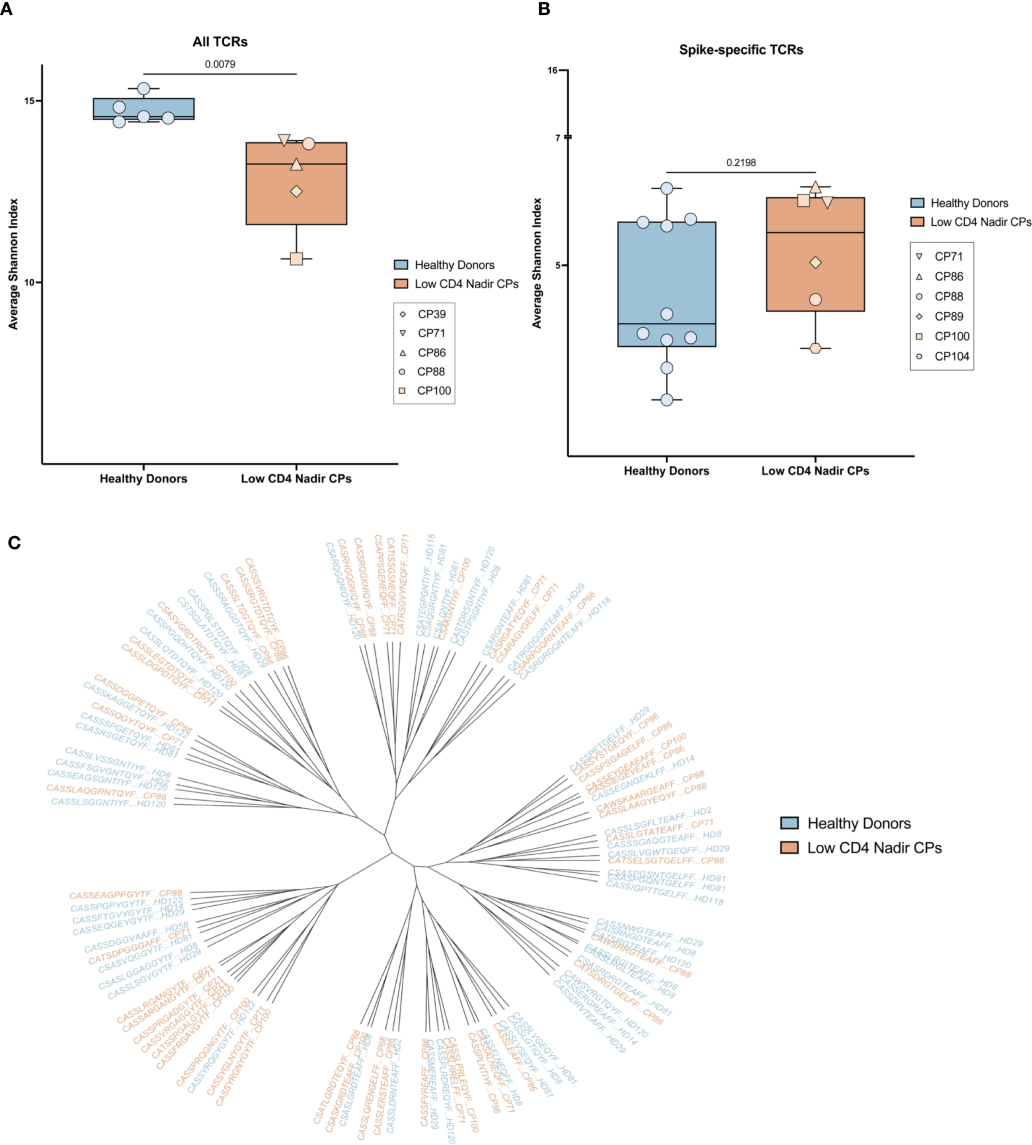
Diversity and sequence homology of T receptors. **(A)** Shannon index of the diversity of all TCRs from PLWH with low CD4 nadirs (orange) and HIV seronegative donors (blue). **(B)** Shannon index of the diversity of SARS-CoV-2-specific TCRs from PLWH with low CD4 nadirs (orange) and HIV seronegative donors (blue). **(C)** Phylogenetic tree showing SARS-CoV-2-specific TCRs from PLWH with low CD4 nadirs (orange) and HIV seronegative donors (blue).

In order to determine whether the total TCR diversity was different in healthy donors versus PLWH with low CD4 nadirs, we analyzed the CD4 TCR repertoire from 5 age matched individuals from each group and analyzed the CD4+ TCR repertoire. Diversity was measured with the Shannon index. As shown in **Figure 4A**, the TCR repertoire from healthy donors was more diverse than TCR repertoire from the PLWH with low CD4 nadirs. There was no correlation between either the nadir or current CD4 count and the Shannon Index (**Supplementary Figure 3A**).

To investigate whether the spike-specific TCR repertoire was also different in the 2 groups of participants, we compared the previously described SARS-CoV-2 spike-specific T cell repertoire in 4 PLWH with low CD4 nadirs (CP88, CP89, CP100, CP104) and 10 healthy donors who received bivalent ancestral/BA.5 spike COVID mRNA vaccines (11). We also analyzed the spike-specific T cell repertoire from 2 PLWH with low CD4 nadirs in the current study (CP71 and CP86). We found there were no significant differences in the diversity of the spike-specific TCR repertoire in PLWH with low CD4 nadirs and healthy donors (**Figure 4B**). Furthermore, in a prior study of healthy donors, we showed that SARS-CoV-2 spike-specific TCRs share sequence homology within and among participants. We performed a similar analysis and showed that there was significant homology of spike-specific TCRs in patients with low CD4 nadirs and healthy donors (**Figure 4C**). There was no correlation between either the nadir or current CD4 counts and the Shannon Index (**Supplementary Figure 3B**).

## Discussion

In this study, we analyzed TCRs specific for SARS-CoV-2 and the 4 common cold coronaviruses spike proteins in PLWH with low CD4 nadirs who had received monovalent and bivalent COVID mRNA vaccines. These individuals responded well to vaccination with antibody titers and CD4+ T cell responses that were similar to healthy donors. The frequencies of SARS-CoV-2 spike-specific effector and memory T cells in these individuals exceeded the frequencies of effector and memory T cells that recognized spike peptides from common cold coronaviruses. We used the FEST assay to distinguish between TCRs that were mono-reactive for SARS CoV-2 versus those that cross-recognized spike proteins from common cold coronaviruses. We previously validated the FEST assay by transferring cloned TCRs into Jurkat cells and demonstrated that TCRs that were identified as cross-reactive in the FEST assay recognized spike peptides from SARS-CoV-2 and HCoV-NL63, whereas TCRs that were identified as being mono-reactive only recognized spike peptides from SARS-CoV-2 (7).

Disruptions of the TCR repertoire in PLWH with low CD4 nadirs are not completely reversed with antiretroviral therapy (3). This could explain the suboptimal responses to immunization that are generally seen in these individuals (1, 2). A skewed TCR repertoire could potentially lead to antigenic imprinting where there is preferential expansion of pre-existing, cross-reactive T cells. This would be pertinent in recipients of COVID vaccines as cross-reactive TCRs have a lower functional avidity for SARS-CoV-2 spike peptides than mono-reactive T cells (6, 7). We reasoned that if antigenic imprinting was occurring in these patients, the spike-specific T cell response would consist predominantly of TCRs primed against the common cold coronaviruses that cross-reacted with SARS-CoV-2. Instead, we found that in all 4 individuals we analyzed, more than 90% of the total SARS-CoV-2 spike specific TCRs were mono-reactive for SARS-CoV-2. We found a similar phenomenon in healthy donors who were vaccinated after experiencing natural infection in a prior study (10), and here we show that in spite of lower total TCR diversity, the diversity of the spike-specific TCR repertoire in the PLWH with low CD4 nadirs we analyzed is comparable to that of healthy donors. Furthermore, we demonstrated that SARS-CoV-2 spike-specific TCRs share sequence homology within and among healthy donors and PLWH with low CD4 nadirs suggesting similar immune responses in these individuals.

Our study is limited by sample size. We evaluated 4 participants with low CD4 nadirs for cross-reactive TCRs, however, we analyzed large numbers of TCRs for each participant, and we saw the same dramatic finding in each of the 4 participants. It is possible that our assay may not detect low frequency clones that could potentially have been cross-reactive. Our diversity analysis is limited by the fact the PLWH with low CD4 nadirs were older and 2 of the 6 individuals were further removed from the time of vaccination compared to the healthy donors. In spite of this, we saw comparable levels of diversity. Interestingly, there appeared to be lower diversity in the SARS-CoV-2 spike specific T cell responses in a subset of the healthy donors, but larger studies will be needed to confirm this finding. Our results suggest that in spite of having CD4 nadirs as low as 2 cells/ul, PLWH can make robust T cell responses in response to SARS-CoV-2 vaccination that are not due to an expansion of pre-existing cross-reactive TCRs. The high titer of SARS-CoV-2 spike-specific antibodies seen in these individuals is most likely a manifestation of this robust functional CD4+ T cell immune reconstitution. CP100, who had undetectable SARS-CoV-2 specific antibodies despite receiving the first dose of the primary mRNA vaccine series and having prolonged SARS-CoV-2 shedding when he had a CD4 count of 2 cells/ul (15), seroconverted after initiating ART and receiving subsequent mRNA vaccine doses. It will be important to analyze TCR repertoire responses to other vaccines in these participants to determine whether this phenomenon is unique to COVID mRNA vaccine induced T cell responses.

## Data Availability

The original contributions presented in the study are publicly available. This data can be found here: https://doi.org/10.6084/m9.figshare.c.8075320.v1.
