# Supplementary material for: SARS-CoV-2 vaccines induce a diverse spike-specific CD4+ T cell receptor repertoire in people living with HIV with low CD4 nadirs
Source: Front Immunol. 2025 Oct 13;16:1663819. doi: 10.3389/fimmu.2025.1663819 (PMC12554773; doi:10.3389/fimmu.2025.1663819)
Supplement: Supplementary file 1 [file DataSheet1.pdf]

Supplementary figure 1

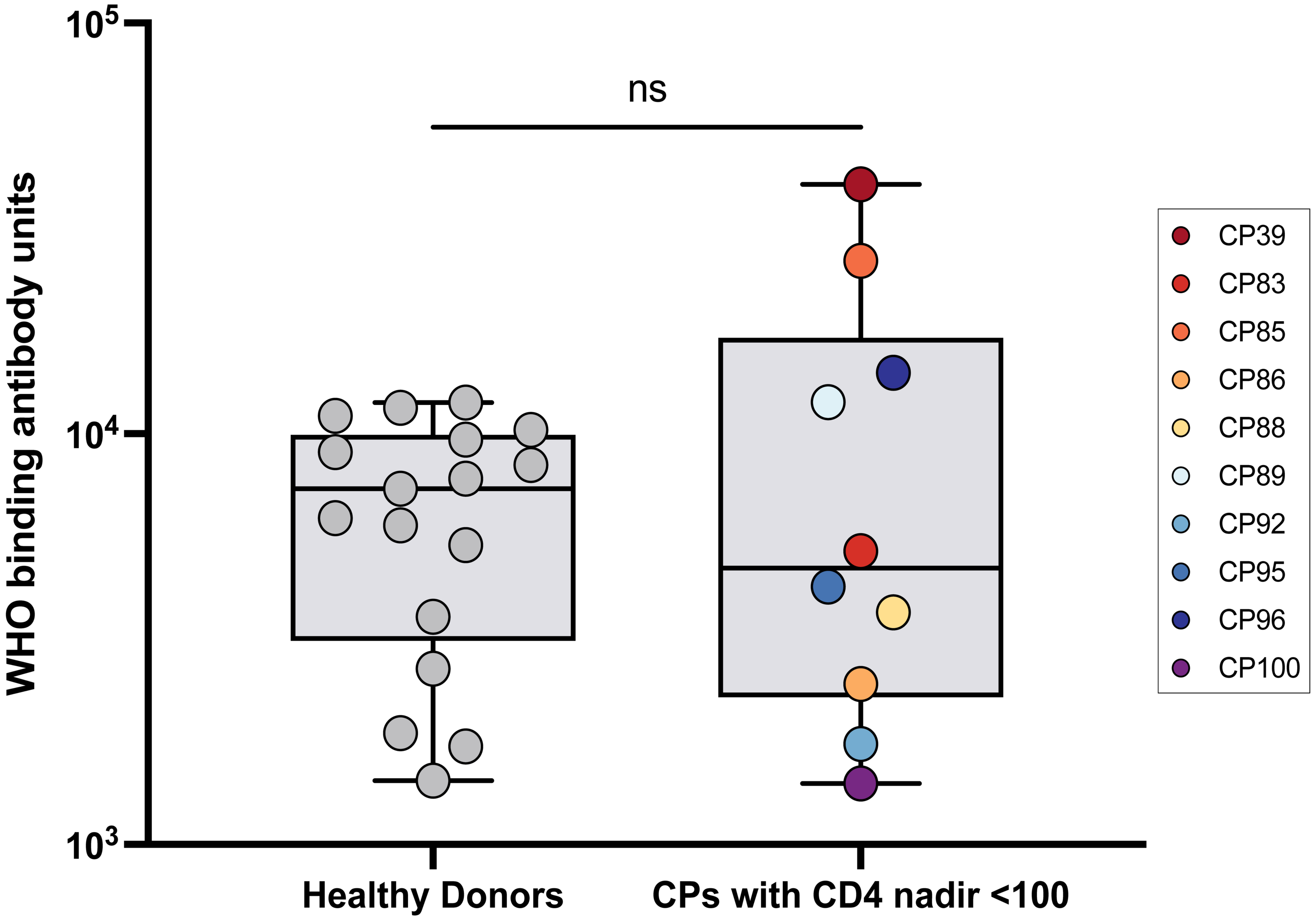

Supplementary Figure 1 Titer of ancestral SARS-CoV-2 spike-binding antibodies by cohort.
Titer of ancestral SARS-CoV-2 spike-binding IgG (WHO binding antibody units, BAU/mL) in HIV-seronegative donors and PLWH with historical CD4 nadir <100.

Supplementary figure 2

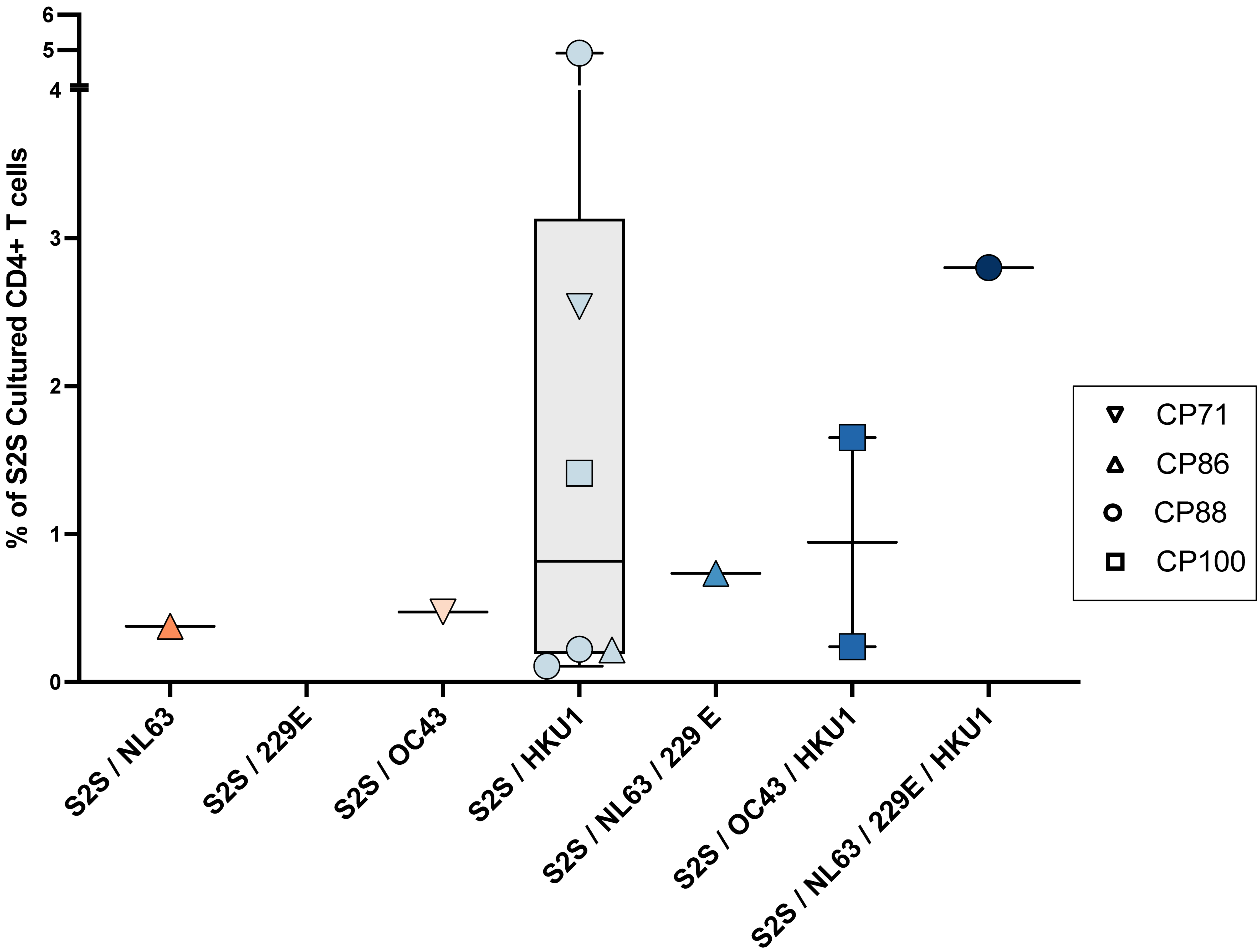

Supplementary Figure 2. Frequency of cross-reactive CD4⁺ T cells among S2S-expanded cultures.

Proportion of cultured CD4⁺ T cells from S2S-stimulated wells that were cross-reactive to additional coronavirus spikes (NL63, 229E, OC43, HKU1) across four CPs. Each point represents the average of three technical replicates for a unique TCR clonotype that expanded in response to S2S and at least one other spike. Combinations on the x-axis indicate the cross-reactivity pattern of each TCR. CP88 had 3 different S2S/HKU1 cross-reactive TCR clonotypes and CP100 had 2 different S2S/OC43/HKU1 cross-reactive TCR clonotypes

Supplementary figure 3

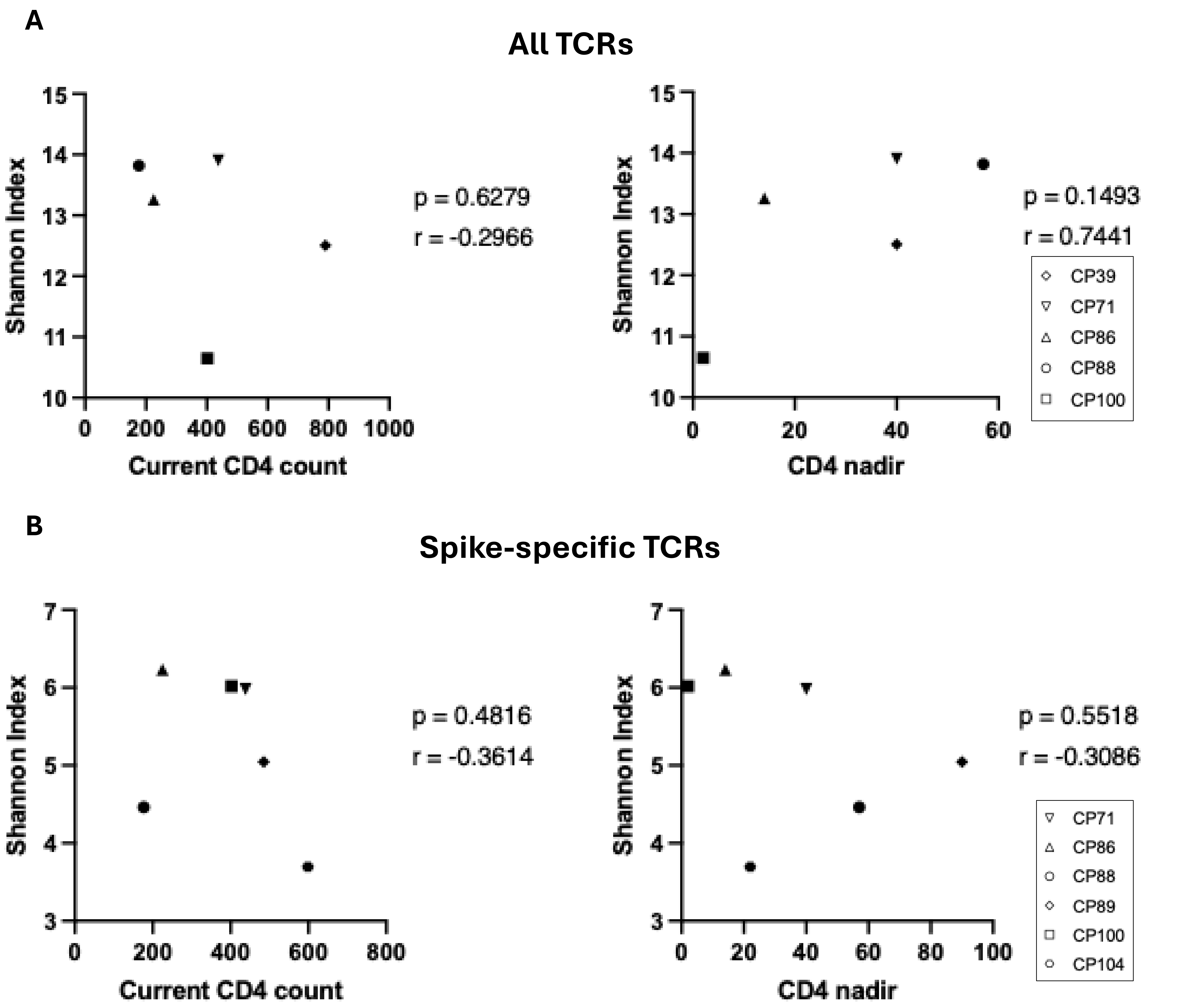

Supplementary Figure 3. Correlation between CD4 counts and TCR repertoire diversity.

(A) All TCRs**.** Shannon index of the bulk TCRβ repertoire plotted against current CD4 count (left) and CD4 nadir (right) for chronic progressors CP39, CP71, CP86, CP88, and CP100. Points = participants (symbols keyed); line = least-squares fit with 95% CI. Stats: Pearson correlation, two-sided; r and p shown on plots.

(B) Spike-specific TCRs**.** Shannon index of SARS-CoV-2 spike–specific TCRβ clonotypes (FEST) versus current CD4 count (left) and CD4 nadir (right) for CP71, CP86, CP88, CP89, CP100, and CP104. Display and statistics as in (A)
