# Supplementary material for: SARS-CoV-2 vaccines induce a diverse spike-specific CD4+ T cell receptor repertoire in people living with HIV with low CD4 nadirs
Source: Front Immunol. 2025 Oct 13;16:1663819. doi: 10.3389/fimmu.2025.1663819 (PMC12554773; doi:10.3389/fimmu.2025.1663819)
Supplement: Supplementary file 2 [file Table1.docx]

Supplementary Table 1. Study participant demographic data (initial enrollment)

| Study  participant | Time between bivalent vaccine and blood draw (days) | Age | Gender | CD4 nadir*  (cells/ul) | Current CD4 count*  (cells/ul) | Years since CD4 nadir | Years HIV RNA < 50 |
| --- | --- | --- | --- | --- | --- | --- | --- |
| CP39 | 153 | 61 | M | 40 | 790 |  |  |
| CP71 | 178 | 63 | M | 40 | 406 | 16 | 15 |
| CP83 | 209 | 62 | M | 32 | 238 |  |  |
| CP85 | 54 | 62 | M | 39 | 141 |  |  |
| CP86 | 154 | 53 | M | 59 | 198 | 5 | 1.5 |
| CP88 | 169 | 57 | M | 50 | 108 |  |  |
| CP89 | 182 | 48 | F | 90 | 485 |  |  |
| CP92 | 196 | 48 | F | 1 | 1721 |  |  |
| CP95 | 231 | 38 | M | 33 | 963 |  |  |
| CP96 | 91 | 37 | M | 28 | 360 |  |  |
| CP100 | 137 | 46 | M | 2 | 224 |  |  |
| CP104 | 307 | 59 | M | 36 | 383 |  |  |
| HD2 | 183 | 46 | M |  | 601 |  |  |
| HD7 | 127 | 53 | F |  |  |  |  |
| HD8 | 189 | 56 | M |  |  |  |  |
| HD14 | 218 | 32 | F |  |  |  |  |
| HD15 | 278 | 43 | M |  |  |  |  |
| HD26 | 188 | 29 | F |  |  |  |  |
| HD29 | 231 | 57 | F |  |  |  |  |
| HD31 | 176 | 43 | M |  |  |  |  |
| HD36 | 177 | 44 | M |  |  |  |  |
| HD39 | 253 | 56 | M |  |  |  |  |
| HD41 | 114 | 47 | M |  |  |  |  |
| HD54 | 212 | 37 | F |  |  |  |  |
| HD58 | 344 | 28 | M |  |  |  |  |
| HD81 | 129 | 24 | F |  |  |  |  |
| HD83 | 289 | 37 | F |  |  |  |  |
| HD117 | 144 | 24 | F |  |  |  |  |
| HD118 | 92 | 32 | F |  |  |  |  |
| HD120 | 54 | 22 | F |  |  |  |  |
| HD121 | 164 | 50 | M |  |  |  |  |
| HD122 | 216 | 34 | M |  |  |  |  |
| HD123 | 232 | 33 | F |  |  |  |  |
| HD126 | 199 | 53 | F |  |  |  |  |
