# Supplementary material for: SARS-CoV-2 vaccines induce a diverse spike-specific CD4+ T cell receptor repertoire in people living with HIV with low CD4 nadirs
Source: Front Immunol. 2025 Oct 13;16:1663819. doi: 10.3389/fimmu.2025.1663819 (PMC12554773; doi:10.3389/fimmu.2025.1663819)
Supplement: Supplementary file 3 [file Table2.docx]

Supplementary Table 2. Study participant demographic data (common cold coronavirus FEST study)

| Study  participant | Time between bivalent vaccine and blood draw (days) | Age | Gender | CD4 nadir*  (cells/ul) | Current CD4 count*  (cells/ul) | Years since CD4 nadir | Years HIV RNA < 50 |
| --- | --- | --- | --- | --- | --- | --- | --- |
| CP71 | 487 | 64 | M | 40 (2%) | 438 (22%) | 16 | 15 |
| CP86 | 446 | 54 | M | 14 (4%) | 225 (18%) | 5 | 1.5 |
| CP88 | 426 | 58 | M | 57 (6%) | 177 (13%) | 4 | 4 |
| CP100 | 467 | 46 | M | 2 (0.4%) | 402 (14%) | 2 | 1.5 |
